# Oxidative Inactivation of Mitochondrial Aconitase Results in Iron and H_2_O_2_-Mediated Neurotoxicity in Rat Primary Mesencephalic Cultures

**DOI:** 10.1371/journal.pone.0007095

**Published:** 2009-09-18

**Authors:** David Cantu, Jerome Schaack, Manisha Patel

**Affiliations:** 1 Neuroscience Program, University of Colorado Denver, Anschutz Medical Campus, Aurora, Colorado, United States of America; 2 Department of Microbiology, University of Colorado Denver, Anschutz Medical Campus, Aurora, Colorado, United States of America; 3 Department of Pharmaceutical Sciences, University of Colorado Denver, Anschutz Medical Campus, Aurora, Colorado, United States of America; Newcastle University, United Kingdom

## Abstract

**Background:**

Mitochondrial oxidative stress is a contributing factor in the etiology of numerous neuronal disorders. However, the precise mechanism(s) by which mitochondrial reactive oxygen species (ROS) modify cellular targets to induce the death of neurons remains unknown. The goal of this study was to determine if oxidative inactivation of mitochondrial aconitase (m-aconitase) resulted in the release of redox-active iron (Fe^2+^) and hydrogen peroxide (H_2_O_2_) and whether this contributes to cell death.

**Methodology/Principal Findings:**

Incubation of rat primary mesencephalic cultures with the redox cycling herbicide paraquat (PQ^2+^) resulted in increased production of H_2_O_2_ and Fe^2+^ at times preceding cell death. To confirm the role of m-aconitase as a source of Fenton reagents and death, we overexpressed m-aconitase using an adenoviral construct thereby increasing the target available for inactivation by ROS. Co-labeling studies identified astrocytes as the predominant cell type expressing transduced m-aconitase although neurons were identified as the primary cell type dying. Oxidative inactivation of m-aconitase overexpressing cultures resulted in exacerbation of H_2_O_2_ production, Fe^2+^ accumulation and increased neuronal death. Increased cell death in m-aconitase overexpressing cultures was attenuated by addition of catalase and/or a cell permeable iron chelator suggesting that neuronal death occurred in part via astrocyte-derived H_2_O_2_.

**Conclusions:**

These results suggest a role of ROS-sensitive m-aconitase as a source of Fe^2+^ and H_2_O_2_ and as a contributing factor to neurotoxicity.

## Introduction

Mitochondrial oxidative stress is a contributing factor in the etiology of numerous chronic and acute neuronal disorders including Parkinson's disease (PD), amyotrophic lateral sclerosis and stroke [1], [2], [3], [4]. However, the precise mechanism(s) by which reactive oxygen species (ROS) modify cellular targets to induce brain injury are not completely understood.

Several important metabolic enzymes are particularly sensitive to ROS such as the iron-sulfur (Fe-S) containing aconitases [5] and the α-ketoglutarate dehydrogenase complex [6]. Mammalian aconitase, like several bacterial dehydratases, contains a [4Fe-4S] prosthetic group which is susceptible to inactivation by superoxide radical (O_2_ **^.^** ^−^) [5], [7], [8]. Aconitase is uniquely sensitive to O_2_ **^.^** ^−^ mediated oxidative inactivation because of the presence of a single unligated iron atom, such that oxidation of the [4Fe-4S]^2+^ promotes cluster instability and consequent loss of the labile iron atom and formation of H_2_O_2_ (Equation 1). Release of redox-active iron (Fe^2+^) from aconitase and other hydro-lyases has been previously reported in cell-free systems [7], [9]. Importantly, purified mitochondrial aconitase (m-aconitase) isolated from bovine heart has been shown to be a source of hydroxyl radical formation (**^.^**OH), presumably via Fenton chemistry initiated by the co-released Fe^2+^ and hydrogen peroxide (H_2_O_2_) [10] (Equation 2).
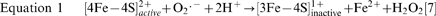


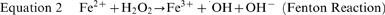



The mitochondria are a major source of ROS where an estimated 0.4–1% of total oxygen consumed in this vital organelle is reduced to O_2_ **^.^** ^−^ [11], [12], [13]. In eukaryotes, two isozymes of aconitase exist; one localized to the matrix of the mitochondria and the other in the cytosol (also known as iron regulatory protein 1). M-aconitase catalyzes the reversible isomerization of citrate and isocitrate via its intermediate form, *cis*-aconitate, in the tricarboxylic acid (TCA) cycle. Because of m-aconitase's unique [4Fe-4S]^2+^ cluster which contains a labile iron atom, and its proximity to mitochondrially generated ROS, it is an ideal candidate for oxidative inactivation. Indeed several neurodegenerative diseases in which oxidative stress has been implicated, as well as *in vivo* and *in vitro* models of these disorders collectively demonstrate decreased aconitase activity [1], [14], [15], [16], [17], [18], [19], [20].

Aconitase has been well established as a sensitive target of ROS; however the consequences of oxidative inactivation of this important enzyme still remain to be fully understood. The role of aconitase(s) in O_2_ **^.^** ^−^ toxicity has been demonstrated in bacteria [8] and yeast [21]. This is based on the premise that oxidation of its [4Fe-4S]^2+^ cluster by O_2_ **^.^** ^−^ in the presence of protons results in the formation of Fe^2+^ and H_2_O_2_ and in turn produce **^.^**OH via Fenton chemistry (Equation 1) [22]. Understanding the role of m-aconitase as a source of ROS in neuronal injury may provide a mechanism by which oxidative modification of ROS-sensitive targets leads to neurodegeneration.

Numerous studies have established a role for iron and mitochondrial oxidative stress as important etiological factors in neurodegenerative disorders such as PD [23], [24], [25]. We have previously shown that m-aconitase is oxidatively inactivated in the 1-methyl-4-phenyl-1,2,3,6-tetrahydropyridine (MPTP) mouse model of parkinsonism and that this correlates with an increase in chelatable mitochondrial iron in the ventral midbrain region [16]. However, whether oxidative inactivation of m-aconitase is a source of Fe^2+^ and H_2_O_2_ in intact neuronal cells and whether it contributes to neurotoxicity remains unknown. The goal of this study was to test the hypothesis that oxidative inactivation of m-aconitase and consequent release of the Fenton reactants H_2_O_2_ and Fe^2+^ contributes to neuronal death. The bipyridyl herbicide paraquat (PQ^2+^) was used to increase steady-state levels of ROS in primary midbrain cultures. PQ^2+^ is a redox cycling compound that produces ROS by a mechanism that involves enzymatic reduction to its cationic radical (PQ^+**.**^) which then reduces molecular oxygen (O_2_) to O_2_ **^.^** ^−^ while also generating the parent compound (PQ^2+^). O_2_ **^.^** ^−^ formed in this manner can increase H_2_O_2_ by at least two mechanisms: 1) dismutation (spontaneous and/or catalysed by superoxide dismutase (SOD) [26] and 2) its reaction with Fe^2+^ from the [4Fe-4S]^2+^ cluster of m-aconitase [22]. Here, we provide evidence that m-aconitase plays a significant role in death of neurons from primary ventral mesencephalic cultures. Using PQ^2+^ as a means of elevating O_2_ **^.^** ^−^, we demonstrate that m-aconitase-dependent increases in H_2_O_2_ and Fe^2+^ contribute to neurotoxicity.

## Methods

### Primary Ventral Mesencephalic Cell Culture

Mixed neuronal and glial cultures were prepared from embryonic day 15–16 (E15–16) rat mesencephalon (Sprague-Dawley, Harlan) according to methods described previously for cortical cultures [17]. Briefly, tissue was dissected as described by Grammatopoulos et al. [27] and enzymatically dissociated using HBSS supplemented with 10 mM HEPES and 2.5% trypsin for 25 min at 37°C. The cells were plated at a density of 80,000 cells/well in poly-D-lysine coated 48-well plates for H_2_O_2_ assay, chamber slides for iron and cell death detection and glass coverslips for immunocytochemistry. Medium was not replaced in order to reduce neuronal loss and glial overgrowth. Approximately one week old midbrain cells (6–8 days in vitro) were used for all experiments. Animal procedures have been reviewed and approved by the University of Colorado Denver Institutional Animal Care and Use Committee. Care was taken to minimize animal suffering and pain.

### Mitochondrial Aconitase Overexpression

M-aconitase was overexpressed in mature primary midbrain cultures using an adenoviral vector. Cells were transduced at a multiplicity of infection (MOI) of 100 plaque forming units (pfu)/cell and incubated at 37°C for 24–48 hrs. Cultures were transduced with an adenoviral construct expressing rat m-aconitase cDNA containing a green fluorescent protein (GFP) reporter (AdAcon). As a control, a separate construct expressing GFP alone (AdGFP) was used. The constructs were assembled with the assistance of the viral vector core facility of the Neuroscience Program at The University of Colorado Denver, Anschutz Medical Campus. Briefly, the pAdTrack-CMV shuttle plasmid was digested with the restriction enzymes XbaI and NotI and rat m-aconitase cDNA (accession NM_024398) was inserted. This placed m-aconitase under the control of the cytomegalovirus major immediate early (CMV) promoter, while a second copy of the CMV promoter controlled expression of GFP which was used as a reporter. The plasmid was then linearized by digestion with PmeI and electroporated into E. coli strain BJ5183 which contained pAdEasy-1. This strain is wild type for RecA allowing for homologous recombination and deleted for the exonuclease (greatly increasing the half life of the linearized shuttle plasmid). After homologous recombination, colonies were grown in the presence of kanamycin (pAdTrack-CMV carries kanamycin resistance). Colonies were screened for the presence of the adenoviral chromosome and the m-aconitase cDNA. To generate virus, the adenoviral vector was liberated from the recombinant plasmid by digestion with PacI and transfected into human embryonic kidney (HEK293) cells. The virus was tested for its ability to overexpress m-aconitase by Western blot analysis (data not shown), then grown in large scale and purified by CsCl gradient centrifugation.

### Real Time PCR Analysis

RNA was extracted from cells using the RNeasy kit® (Qiagen, USA) and quantified using the RiboGreen® RNA Quantitation Kit (Molecular Probes, Eugene, OR) as described by manufacturers' instructions. Real time PCR was performed on an Applied Biosystems 7500 Fast Real-Time PCR System. RNA was reverse transcribed to cDNA using the High-Capacity cDNA Reverse Transcription Kit (Applied Biosystems). Thermal cycling conditions included 25°C for 10 min, 37°C for 120 min and 85°C for 5 sec; samples were stored at 4°C. The PCR reaction conditions used were 1 cycle at 50°C for 2 min, 1 cycle at 95°C for 10 min and 40 cycles of 95°C for 15 sec and 60°C for 1 min. The primers and probes for rat m-aconitase were purchased from Integrated DNA Technologies; forward primer: 5′ CCG CCT TCC TGT TCA GTT TG-3′, reverse primer: 5′ TGT AGA GGG AGT GCT GTC ATC AA-3′.

### Detection of H_2_O_2_

H_2_O_2_ was measured using Amplex Red (Invitrogen, Carlsbad, CA), a horse radish peroxidase (HRP)-linked fluorometric assay. Cell culture media was removed and replaced with 250 µl of a Hank's Buffered Saline Solution (HBSS) solution containing 1 mg/ml glucose, 0.1 U/ml HRP, and 50 µM Amplex Red. The reaction was started with the addition of 250 µM, 500 µM or 1000 µM PQ^2+^ (final concentration) to the HBSS solution. Resorufin fluorescence was measured 2, 4, and 6 hrs after cell treatment by a Gemini fluorescence microplate reader equipped for excitation in the range of 530–560 nm and fluorescence emission detection at 590 nm (Molecular Devices, Sunnyvale, CA).

### Mitochondrial Fe^2+^ detection

Detection of free mitochondrial iron was conducted using rhodamine B-[(1,10)phenanthrolin-5-yl) aminocarbonyl]benzyl ester (RPA) staining [28], a fluorescent iron indicator whose fluorescence is quenched by iron. Midbrain cells were incubated with 250 µM, 500 µM and 1000 µM PQ^2+^ (final concentration) added directly to the cell culture media for 4 hrs. Cell culture media was then removed and replaced with 250 µL of 1 µM RPA dissolved in HBSS. Cells were kept at 37°C for 10 min, rinsed with HBSS, and placed at 37°C for an additional 10 min. Cultures were rinsed a final time with HBSS before 3 randomly selected images were captured on an Olympus IX81 inverted motorized microscope. Images were quantified by measuring the mean pixel intensity using ImageJ (NIH).

### Cell Treatment

For experiments designed to test the role of H_2_O_2_ or iron, catalase (100 U/mL) (Sigma) and N,N′-bis (2-hydroxybenzyl) ethylenediamine-N,N′-diacetic acid (HBED) (50 µM) (Strem Chemicals) were added 1 hr prior to the addition of PQ^2+^. Mitochondrial localization of RPA was performed with a mitochondrial marker, 3,3′-dihexyloxacarbocyanine iodide (DiOC_6_(3)) (Invitrogen). Immediately after RPA staining, HBSS was removed and replaced with 250 µL of 100 nM DiOC_6_(3) dissolved in HBSS. Cells were kept at 37°C for 10 min, rinsed with HBSS, and placed at 37°C for an additional 10 min. Cultures were rinsed a final time with HBSS before images were captured on an Olympus IX81 inverted motorized microscope.

### Mitochondrial Dysfunction and Cell Death Assessment

Loss of cell viability due to mitochondrial dysfunction was analyzed using 3-(4,5-Dimethyl-2-thiazolyl)-2,5-diphenyl-2H-tetrazolium bromide (MTT) (Sigma). Cells were incubated with 5 mg/ml MTT added directly to the cell culture media for 15 min. Cells were washed with PBS and formazan crystals were dissolved with isopropanol containing 4 mM hydrochloric acid. Absorbance measurements were taken at 590 nm with a Versamax microplate reader (Molecular Devices). Detection of cell death was performed using Propidium Iodide (PI) staining (Invitrogen, Eugene, OR). Cells were rinsed with 2X SSC (0.3M NaCl, 0.03M sodium citrate, pH 7.0), fixed with 4% paraformaldehyde for 30 min, then washed again with 2X SSC. This was followed by DNase-free RNase (100 µg/ml in 2X SSC) incubation for 20 min at 37°C, and rinsed once again with 2X SSC. Cells were then incubated in 2 µM PI for 5 min, and washed with 2X SSC. 3 randomly selected images were captured using an Olympus IX 81 inverted motorized microscope and PI+ cells were counted.

### Aconitase and Fumarase Activities

Aconitase and fumarase activities were measured spectrophotometrically as previously described [17] with minor modifications including increasing the lag time to 5 min and decreasing the total sample size to 500 µL from 1 mL.

### Immunocytochemistry

Cells were fixed in 4% paraformaldehyde in phosphate buffered saline (PBS) for 15 min, washed with PBS and blocked in carrier solution (0.3% Triton-X, 1% BSA in PBS) containing 10% normal goat serum (NGS) for 1 hr. The following primary antibodies were diluted in carrier solution containing 1% NGS and added overnight at 4°C: microtubule associated protein 2 (MAP2) (1∶250), glial acidic fibrillary protein (GFAP) (1∶500). Cultures were washed again in PBS and appropriate secondary antibody (goat anti-mouse or –rabbit conjugated to a rhodamine fluorophore) was added (1∶1000) for 2 hrs at room temperature. DAPI (1 µg/mL) was used as a counter stain to identify nuclei. Coverslips were then mounted on glass slides using an anti-fading agent (0.1% *p*-Phenylenediamine, 50% glycerol in PBS) and imaged using an Olympus IX81 inverted motorized microscope.

### Statistical Analysis

For comparison between three or more experimental groups, one-way ANOVA with the Bonferroni post hoc test was used. A two-way ANOVA was used for comparing AdGFP vs. AdAcon at multiple doses of PQ^2+^. Values of *p<0.05 or more were considered statistically significant.

## Results

To address the role of m-aconitase as a source of H_2_O_2_ and Fe^2+^, we asked (i) whether oxidative inactivation of m-aconitase by PQ^2+^ resulted in accumulation of H_2_O_2_, Fe^2+^ and cell death in primary midbrain cultures, (ii) if overexpressing m-aconitase exacerbated these effects, (iii) whether cell death could be prevented via removal of Fenton reagents using an iron chelator and antioxidant and (iv) whether astrocytes and/or neurons were dying.

### H_2_O_2_ production, mitochondrial Fe^2+^ accumulation and cell death occur following oxidative inactivation of aconitase

**Figure 1:**
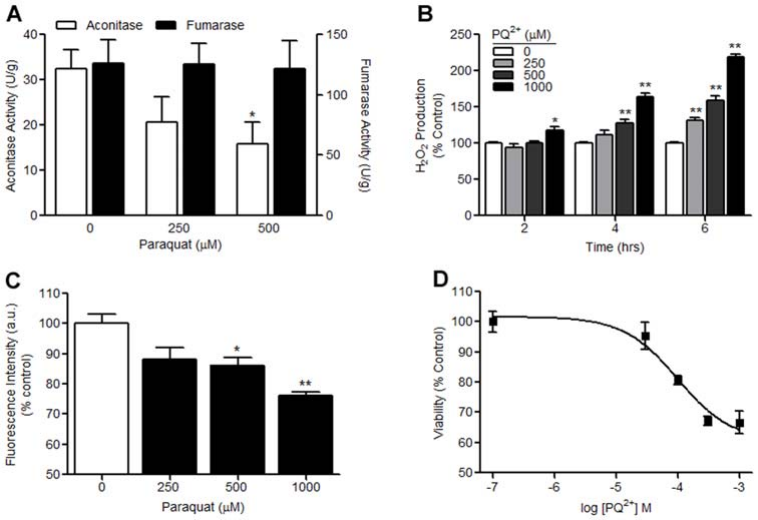
Aconitase activity, H_2_O_2_ production, mitochondrial Fe^2+^ and cell viability in PQ^2+^ treated primary midbrain cultures. (A) Primary midbrain cells were treated with 0, 250 and 500 µM PQ^2+^ for 3 hrs and aconitase and fumarase activities were measured spectrophotometrically. Data are expressed as units/grams of protein, bars represent mean±SEM, *p<0.05, one-way ANOVA (n = 3–4). (B) Primary midbrain cultures were incubated with 0, 250, 500 and 1000 µM PQ^2+^. H_2_O_2_ production was measured by amplex red after 2, 4 and 6 hrs. Data are expressed as % control; asterisks indicate a difference from vehicle treated control at each time point. Bars represent mean±SEM, *p<0.05, **p<0.001, two-way ANOVA (n = 12). (C) Mitochondrial Fe^2+^ was measured by RPA fluorescence. Mean pixel intensity of 3 random fields/well was quantified using Image J (NIH) and expressed as % control. Asterisks indicate difference from vehicle treated controls. Bars represent mean±SEM, *p<0.05, **p<0.01, one-way ANOVA (n = 6). (D) Cell viability was assessed spectrophotometrically using MTT after 18 hrs of PQ^2+^ incubation. Each point represents mean±SEM (n = 5).

We first determined the effect of the O_2_ **^.^** ^−^ generating compound PQ^2+^ on aconitase activity, H_2_O_2_ production, mitochondrial Fe^2+^ accumulation and cell death in primary midbrain cultures. Consistent with our previous work [17] aconitase activity was decreased after 2–3 hrs of PQ^2+^ incubation while the activity of fumarase, a control enzyme which lacks an oxidation sensitive Fe-S center, remained unchanged suggesting a role for oxidative stress in the mechanism of aconitase inactivation (Fig. 1a). A time- and concentration-dependent increase in H_2_O_2_ production was observed in midbrain cultures following PQ^2+^ addition. A significant increase in H_2_O_2_ production was detected after 2 hrs of incubation with 1000 µM PQ^2+^ (117±5.2%), 4 hrs of incubation with 500 µM and 1000 µM PQ^2+^ (128±4.6% and 164±4.2%, respectively) and 6 hrs of 250 µM, 500 µM and 1000 µM PQ^2+^ incubation (131±4.2%, 159±6.2% and 219±3.7%, respectively) (Fig. 1b). To detect changes in mitochondrial Fe^2+^ we used RPA fluorescence whereby a decrease in fluorescence indicates an increase in Fe^2+^. A concentration-dependent decrease in RPA fluorescence (corresponding with an increase in Fe^2+^) was detected following 4 hrs of incubation with 500 µM (13.8±2.4%) and 1000 µM (23.8±1.2%) PQ^2+^ (Fig. 1c). Additionally, a concentration-dependent decrease in cell viability following 18 hrs of PQ^2+^ incubation was detected (Fig. 1d). Together these studies demonstrate increased H_2_O_2_ production and mitochondrial iron accumulation following oxidative inactivation of aconitase at times preceding cell death.

### Transduction with AdAcon increases aconitase expression and activity in primary midbrain cultures

**Figure 2:**
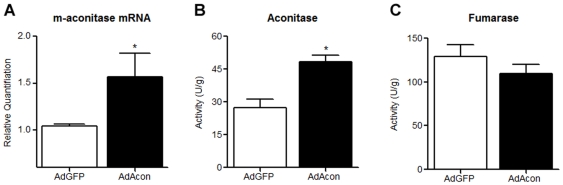
Aconitase expression and activity are increased with AdAcon transduction. Cells were transduced with AdGFP and AdAcon for 24–48 hrs. (A) m-aconitase mRNA was measured by real time PCR. Aconitase (B) and fumarase (C) activities were measured spectrophotometrically, bars represent mean±SEM, *p<0.05, t-test (n = 3–6).

To confirm the role of m-aconitase versus other cellular proteins in the production of Fe^2+^ and H_2_O_2_, we specifically overexpressed m-aconitase. We hypothesized that increasing the levels of m-aconitase would increase the amount of target enzyme available for oxidative inactivation leading to exacerbation of H_2_O_2_ production, Fe^2+^ formation and cell death. Overexpression of m-aconitase was achieved by transducing cells with an adenoviral construct expressing m-aconitase cDNA and a GFP reporter (AdAcon) or with an adenoviral construct only expressing GFP (AdGFP) to control for viral-mediated effects. Successful transduction was observed between 24–48 hrs via fluorescent detection of GFP. To confirm that m-aconitase levels were indeed increased, m-aconitase mRNA from AdAcon transduced cells was compared to cells transduced with AdGFP. Cells transduced with AdAcon showed a significant increase in m-aconitase mRNA (∼50%) compared to AdGFP control 24 hrs post-transduction (Fig. 2a). Additionally, aconitase activity increased after 24–48 hrs in cells transduced with AdAcon compared to AdGFP transduced cells while the activity of fumarase remained unchanged (Fig. 2b,c).

### Oxidative inactivation of overexpressed m-aconitase exacerbates H_2_O_2_ production and mitochondrial iron accumulation

**Figure 3:**
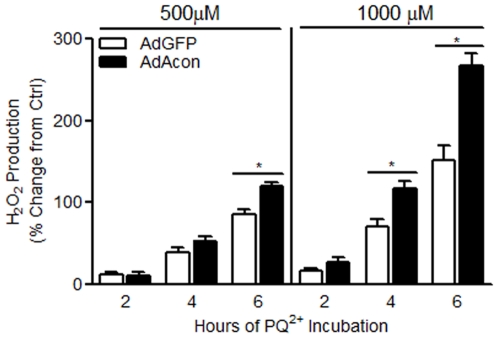
M-aconitase overexpressing cells produce higher levels of H_2_O_2_ in the presence of PQ^2+^. Primary midbrain cultures were transduced with AdGFP and AdAcon for 24–48 hrs and treated with 0, 500 and 1000 µM PQ^2+^. H_2_O_2_ was measured at 2, 4 and 6 hrs using amplex red. Data are expressed as % increase of vehicle treated AdGFP transduced cells. Bars represent mean±SEM, *p<0.05, two-way ANOVA, (n = 7–14).

We proceeded to test whether increasing m-aconitase expression would exacerbate the release of Fenton reactants following oxidative inactivation. Primary midbrain cultures transduced with AdAcon demonstrated higher levels of H_2_O_2_ production compared to AdGFP transduced cells at 6 hrs of 500 µM as well as 4 and 6 hrs of 1000 µM PQ^2+^ incubation (Fig. 3). As expected, PQ^2+^ alone increased H_2_O_2_ production; however, this was significantly exacerbated when m-aconitase was overexpressed suggesting the dependence of m-aconitase in the H_2_O_2_ production.

**Figure 4:**
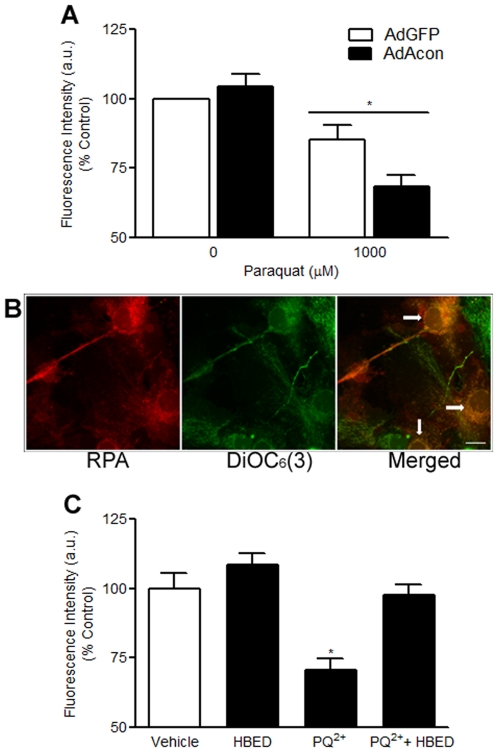
M-aconitase overexpressing cells produce higher levels of mitochondrial Fe^2+^ in the presence of PQ^2+^. (A) Primary midbrain cultures were transduced with AdGFP and AdAcon for 24 hrs, treated with 1000 µM PQ^2+^ and mitochondrial Fe^2+^ was measured by quantifying mean pixel intensity of RPA fluorescence after 4 hrs. Bars represent mean±SEM, *p<0.05, two-way ANOVA, (n = 6). (B) RPA localizes to the mitochondria. Primary midbrain cultures were treated with 1 µM RPA immediately followed by 100 nM DiOC_6_(3) and imaged at 40X as described in experimental procedures. Representative images, arrows indicate co-localization, scale bar = 15 µm. (C) HBED can prevent the release of Fe^2+^ from oxidative inactivation of m-aconitase. M-aconitase overexpressing primary midbrain cultures were pre-treated with HBED for 1 hr, exposed to 1000 µM PQ^2+^ for 4 hrs and stained with RPA. Bars represent mean±SEM, *p<0.05, one-way ANOVA, (n = 7).

As shown in Fig. 1, a concentration-dependent increase in Fe^2+^ was detected in primary midbrain cultures after 4 hrs of PQ^2+^ incubation. To verify whether oxidative inactivation of m-aconitase played a role in releasing Fe^2+^ in addition to H_2_O_2_, m-aconitase was overexpressed with AdAcon and levels of Fe^2+^ were measured. M-aconitase overexpressing cells showed a significant decrease in RPA fluorescence (indicating an increase in free mitochondrial Fe^2+^) after treatment for 4 hrs with 1000 µM PQ^2+^ (Fig. 4a). Similar to the observed increases in H_2_O_2_ in m-aconitase overexpressing cells, Fe^2+^ was increased to greater levels in AdAcon compared to AdGFP transduced cells suggesting that both H_2_O_2_ and Fe^2+^ can be released from oxidatively inactivated m-aconitase.

### Mitochondrial Fe^2+^ release from m-aconitase can be attenuated with HBED

To verify whether oxidative inactivation of m-aconitase resulted in release of Fe^2+^ from mitochondria, we asked whether RPA co-localized with DiOC_6_(3), a mitochondrial marker and if PQ^2+^-induced changes could be prevented with HBED, an iron chelator capable of permeating mitochondria [29]. Double-staining experiments revealed that cells stained with RPA and DiOC_6_(3) strongly co-localized (Fig. 4b) suggesting that the iron detected by the RPA stain was localizing to the mitochondrial compartment. Furthermore, PQ^2+^-induced increase in Fe^2+^ detected by RPA staining in m-aconitase overexpressing cells was inhibited by pre-treatment with HBED (Fig. 4c). Together these studies suggest that inactivation of m-aconitase increases mitochondrial iron.

### Oxidative inactivation of m-aconitase leads to cell death

**Figure 5:**
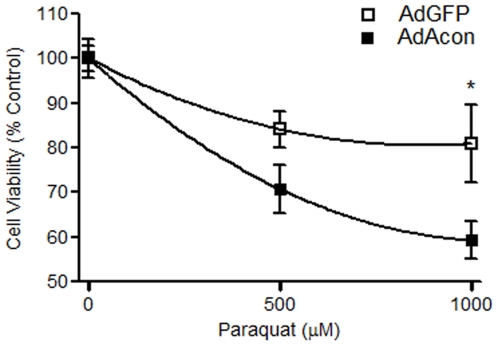
Cells overexpressing m-aconitase show decreased cell viability following PQ^2+^. Primary midbrain cultures were transduced with AdGFP (open squares) and AdAcon (closed squares) for 24 hrs. Cell viability was examined between the two groups after 18 hrs of 0, 500 and 1000 µM PQ^2+^ incubation using the MTT assay. Data are expressed as % cell viability compared to vehicle treated control. Each point represents mean±SEM, *p<0.05, two-way ANOVA, (n = 13).

**Figure 6:**
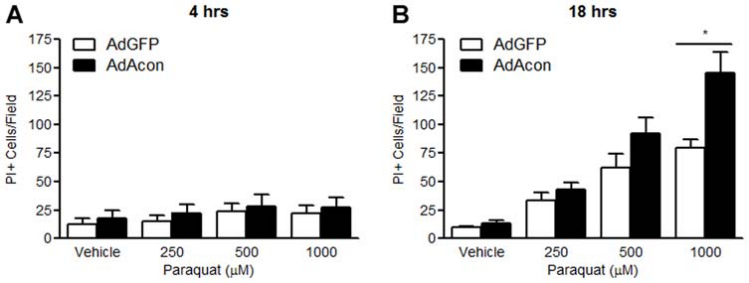
M-aconitase overexpressing cells show increased cell death after PQ^2+^ exposure at times following release of Fenton reactants. Cells were transduced with AdGFP and AdAcon for 24 hrs and incubated with 0, 250, 500 and 1000 µM PQ^2+^. Cells were stained with PI and imaged from 3 randomly selected fields. PI+ cells were counted after (A) 4 hrs of PQ^2+^ incubation and (B) 18 hrs of PQ^2+^ incubation; data are expressed as number of PI+ cells/field. Bars represent mean±SEM, *p<0.05, two-way ANOVA, (n = 5).

One consequence of co-releasing the Fenton ingredients Fe^2+^ and H_2_O_2_ in neuronal cells is cell death; either independently or via the formation of the highly reactive **^.^**OH. We therefore asked whether oxidative inactivation of m-aconitase resulted in mitochondrial dysfunction and cell death 18 hrs after PQ^2+^ incubation in AdGFP vs. AdAcon transduced cells using the MTT assay. Overexpression of m-aconitase resulted in a concentration-dependent decrease of cell viability compared to GFP-only expressing cells (Fig. 5). Cell death was further analyzed by counting PI+ cells after 4 and 18 hrs of PQ^2+^ incubation. We did not detect a difference in PI staining between AdGFP and AdAcon transduced cells treated with PQ^2+^ at 4 hrs (Fig. 6a), suggesting that the observed differences in Fe^2+^ shown in Fig. 4 at this same time point were not due to cell death. However, at a later time point of 18 hrs, when differences in cell viability were detected, m-aconitase overexpressing cells demonstrated a significant increase in PI+ cells in the presence of PQ^2+^ (Fig. 6b) suggesting that delayed cell death was resulting from m-aconitase inactivation. Importantly, the time-course of cell death confirms that H_2_O_2_ formation and Fe^2+^ release occur hours prior to cell death, suggesting that oxidative inactivation of m-aconitase contributes to cell death in primary midbrain cultures.

### Cell death can be prevented by removing H_2_O_2_ and Fe^2+^

**Figure 7:**
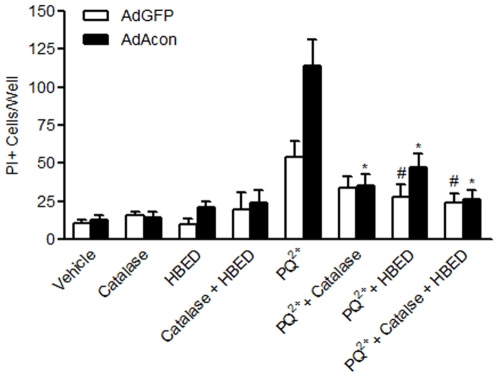
Catalase and/or HBED inhibit PQ^2+^-induced cell death in m-aconitase overexpressing primary midbrain cultures. Cells were pre-incubated with catalase and HBED for 1 hr prior to incubation with 1000 µM PQ^2+^. Cells were stained with PI after 18 hrs of PQ^2+^ incubation. Images of PI+ cells were collected from 3 randomly selected fields and counted; data are expressed as number of PI+ cells/field. Bars represent mean±SEM, *p<0.05 compared to PQ^2+^-treated AdAcon, #p<0.05 compared to PQ^2+^-treated AdGFP, two-way ANOVA, (n = 3–8).

To confirm the mechanism of H_2_O_2_ and Fe^2+^ in the death of cells overexpressing m-aconitase, we asked whether cell death could be prevented by their pharmacological removal using catalase and HBED respectively. Extracellular catalase which is cell impermeable was used as a “sink” for H_2_O_2_ generated within cellular compartments since H_2_O_2_ can cross cellular compartments. In addition, the use of extracellular catalase allowed us to determine any role played by extracellular H_2_O_2_ in the cell death process. In AdGFP transduced cells, HBED alone but not catalase alone inhibited PQ^2+^-induced cell death assessed by PI+ staining after 18 hrs. This is consistent with intramitochondrial Fe^2+^ being the chief mediator of cell death most likely via the Fenton reaction. However, in m-aconitase overexpressing cells, either catalase or HBED was sufficient to significantly inhibit cell death (Fig. 7). The combination of HBED and catalase did not provide further protection compared to HBED alone in either AdGFP or AdAcon transduced cells. The differential effect of catalase between AdGFP vs. AdAcon transduced cells suggests that in addition to the intracellular mechanism (i.e. Fenton reaction); extracellular H_2_O_2_ was also mediating cell death.

### Astrocytic overexpression of m-aconitase results in neuronal death

**Figure 8:**
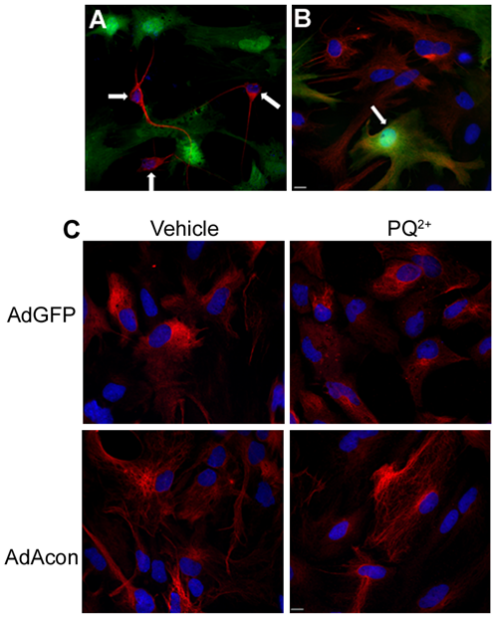
Overexpression of m-aconitase does not lead to cell death of astrocytes. Primary midbrain cultures were transduced with AdAcon for 24–48 hrs and GFP fluorescence (green) was observed. Representative images of (A) neurons detected by MAP2 staining (red) and (B) astrocytes detected by GFAP staining (red). (A,B) Arrows point to neurons and astrocyte, respectively. (C) AdGFP and AdAcon transduced astrocytes were labeled with GFAP (red) after 18 hrs of 1000 µM PQ^2+^ incubation. Nuclei (blue) were counterstained with DAPI, representative images, 40× magnification.

**Figure 9:**
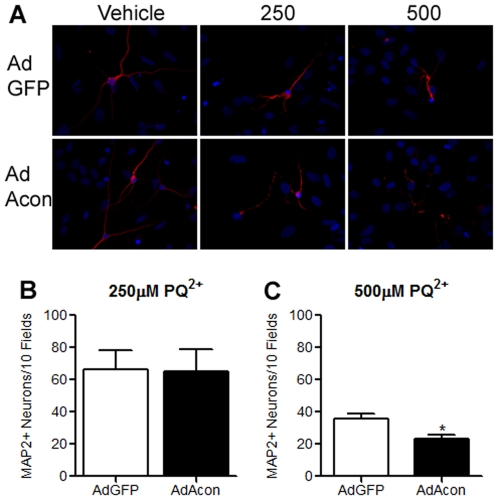
Overexpression of m-aconitase leads to neuronal loss. (A) Representative images of neurons from AdGFP and AdAcon transduced cultures stained with MAP (red) after 18 hrs of 0, 250 and 500 µM PQ^2+^ incubation. Nuclei (blue) were counterstained with DAPI. Neurons with intact nuclei from AdGFP and AdAcon transduced cultures were counted after 18 hrs of 250 µM PQ^2+^ (B) and 500 µM PQ^2+^ (C), data are expressed as MAP2+ neurons/10 fields, * p<0.05, t-test (n = 6).

To determine which cell types in the primary midbrain cultures overexpressed AdAcon, we performed co-localization experiments of GFP with neuron- or astrocyte-specific markers. Co-localization of GFP fluorescence with MAP2 and GFAP performed 24 hrs after transduction with AdAcon, revealed the expression of m-aconitase largely in GFAP positive cells, suggesting that m-aconitase was predominately overexpressed in astrocytes (Fig. 8a,b). Similar results were obtained in AdGFP transduced cells (data not shown). Previous work using mixed neuronal/glial cortical cultures demonstrated earlier and more robust inactivation of aconitase in mixed neuronal/glial cultures vs. near-pure astrocytic cultures [30]. Since adenoviral transduction of m-aconitase predominately resulted in astrocytic expression we determined whether neurons and/or astrocytes were more susceptible to death following m-aconitase inactivation. GFAP staining from AdGFP and AdAcon transduced cultures revealed no change in morphology and no condensation of nuclei after 18 hrs of 1000 µM PQ^2+^ incubation suggesting that cell death was not astrocytic (Fig. 8c). Alternatively, MAP2 staining revealed shortening and thinning of neuronal processes as well as nuclear condensation with increasing concentrations of PQ^2+^ (Fig. 9a). More importantly, neurons from AdAcon transduced cultures revealed greater damage than AdGFP transduced cells at 500 µM PQ^2+^ supporting that m-aconitase overexpression exacerbates neuronal death upon oxidative inactivation. In order to confirm these observations, we counted the neurons from AdGFP transduced cultures and compared them to neurons from AdAcon transduced cultures after 18 hrs of 250 and 500 µM PQ^2+^. Although no difference was detected at 250 µM PQ^2+^ (Fig. 9b), there was a significant decrease in MAP2+ neurons in AdAcon transduced cells compared to AdGFP at 500 µM PQ^2+^ (Fig. 9c). Taken together, this suggests that although astrocytes are predominately overexpressing m-aconitase, neurons are more susceptible to death.

## Discussion

Four major findings in this study provide evidence that m-aconitase is a source of Fenton reactants (H_2_O_2_ and Fe^2+^) in primary midbrain cultures which may be contributing to neurotoxicity. First, treatment of primary midbrain cultures with exogenous oxidative stress via PQ^2+^ resulted in a time- and concentration-dependent increase in H_2_O_2_, Fe^2+^and cell death. Second, PQ^2+^-induced increases in H_2_O_2_, Fe^2+^ and cell death were exacerbated in primary midbrain cells overexpressing m-aconitase. Third, removal of Fenton reactants using a mitochondrial permeable iron chelator and catalase ameliorated cell death. Finally, neurons showed greater vulnerability to oxidative inactivation of m-aconitase. This suggests that in addition to its well known role as a target of ROS, m-aconitase can also be a source of ROS and iron which are neurotoxic.

Decreased aconitase activity is observed in various human neurodegenerative diseases associated with mitochondrial oxidative stress e.g. Huntington's disease, progressive supranuclear palsy, Friedreich's ataxia, and temporal lobe epilepsy [1], [19], [20], [31]. In addition, aconitase inactivation has been observed in various animal and cell models of neuronal disorders and oxidative stress [32], [33] including excitotoxicity [17], cerebral ischemia [34], beta-amyloid toxicity [35], oxygen-glucose deprivation [30], MPTP toxicity [16], Sod2 and DJ1 mutant mice [18], [36], [37] as well as in aging [38], [39]. Aconitase has been widely recognized as a sensitive and relatively specific target of ROS, particularly O_2_ **^.^** ^−^. Both *in vitro* and *in vivo* studies have utilized oxidative inactivation of aconitase as an index of increased ROS levels. In the brain, the levels of m-aconitase predominate allowing the measurement of m-aconitase activity as a surrogate marker of mitochondrial oxidative stress. Collectively, these studies demonstrate that m-aconitase is a sensitive target of ROS generated in cells and tissue.

Work in our laboratory in the MPTP mouse model of parkinsonism suggests that in addition to being a target of ROS, m-aconitase may also be an important source of mitochondrial iron [16]. Although aconitase has been implicated as a source of iron and toxicity in bacteria and yeast [8], [21], its ability to release redox active iron and H_2_O_2_ in neuronal cells and its relationship with neuronal death has not been demonstrated. Our data demonstrate that oxidative inactivation of m-aconitase results in the formation of Fenton reactants in neuronal cells which provides evidence for the pathogenic mechanism in which m-aconitase not only serves as a target but also as a source of oxidants. The role of purified aconitase as a source of **^.^**OH from H_2_O_2_ and Fe^2+^ was first suggested in cell free systems by Flint et al. (1993) based on its reaction with O_2_ **^.^** ^−^, the lability of the Fe_α_ and the unstable cubane Fe-S cluster (Equations 1,2). Evidence of **^.^**OH formation in cell free systems, presumably via the release of Fenton reactants by oxidative inactivation of m-aconitase was provided by Vasquez-Vivar et al. (2000). PQ^2+^-induced H_2_O_2_ production in AdGFP expressing cultures most likely occurs by a combination of O_2_ **^.^** ^−^ dismutation and oxidation of the [4Fe-4S]^2+^ cluster of m-aconitase. Exacerbation of PQ^2+^-induced H_2_O_2_ production observed in m-aconitase overexpressing cultures presumably originates from the latter source due to increased m-aconitase available for inactivation by O_2_ **^.^** ^−^ (Equation 1). The exacerbation of Fe^2+^ and H_2_O_2_ production in m-aconitase overexpressing cells suggests its role as their source.

Two findings suggest that the increased production of H_2_O_2_ and Fe^2+^ in m-aconitase overexpressing cells resulted in mitochondrial dysfunction and neurotoxicity. First, the production of H_2_O_2_ and Fe^2+^ was detected between 4–6 hrs and preceded mitochondrial dysfunction assessed by the MTT assay and cell death assessed by PI staining which were detectable only after 18 hrs of incubation with PQ^2+^. Secondly, scavenging Fe^2+^, a key ingredient of the Fenton reaction with a cell permeable iron chelator provided neuroprotection in both AdGFP and AdAcon transduced cells. The formation of Fenton reactants several hours prior to the onset of cell death and their pharmacological removal, strongly suggests their role in neurotoxicity.

Several lines of evidence suggest that the iron changes reported herein originated from the mitochondrial compartment. First, RPA has been well defined as a fluorescent indicator specific to the mitochondrial compartment [28]. Further proof that the iron originated from the mitochondrial compartment comes from the use of HBED, a cell permeable iron chelator capable of permeating the mitochondria [29]. Accordingly, treatment of cells with HBED attenuated the decrease in RPA fluorescence. In addition, co-localization of RPA with the mitochondrial marker DiOC_6_(3) confirmed its localization to the mitochondria. Finally, confirmation of mitochondrial dysfunction using the MTT assay further suggested mitochondrial formation of Fenton reactants. Collectively, the results are consistent with intramitochondrial Fe^2+^ being the chief mediator of neuronal death most likely via the Fenton reaction.

Intramitochondrial Fe^2+^ in conjunction with H_2_O_2_ via the Fenton reaction may be the chief mediator of neuronal death following oxidative inactivation of endogenous m-aconitase residing in both neurons and astrocytes. This is based on the ability of HBED but not catalase to significantly inhibit cell death in AdGFP transduced cultures. The use of cell impermeable extracellular catalase not only provided a sink for ROS generated within cellular compartments, but revealed a key role of astrocyte-derived extracellular H_2_O_2_ in the death of neurons in m-aconitase overexpressing cultures. The ability of catalase to inhibit neuronal death in AdAcon to a greater extent than AdGFP transduced cells, suggests that in addition to the intracellular mechanism (i.e. Fenton reaction) in cells expressing basal levels of m-aconitase, extracellular H_2_O_2_ was also mediating cell death in m-aconitase overexpressing cultures. Since AdAcon was primarily transduced in astrocytes and cell death was evident in neurons, but not astrocytes, it suggests that oxidative inactivation of astrocytic m-aconitase results in H_2_O_2_ release which kills neurons in a paracrine manner. In summary, since catalase can only attenuate cell death when m-aconitase is overexpressed, this suggests 1) that the excess H_2_O_2_ from m-aconitase inactivation is being released from astrocytes and injuring neighboring neurons and 2) that removal of astrocyte-derived extracellular H_2_O_2_ can prevent death. Of note, the use of PQ^2+^ as a toxicant was particularly advantageous in our model because in contrast to neuron-specific toxicants such as MPP+, PQ^2+^ is capable of inactivating m-aconitase in both mixed neuronal/glial cultures and near pure astrocytic cultures [30].

Although astrocytes were the predominant cell type to overexpress m-aconitase, the neuronal population was more susceptible to death compared to astrocytes. This became apparent by the difference in number of MAP2+ neurons between cultures expressing basal levels of m-aconitase and those overexpressing the enzyme as well as by lack of damage to astrocytic morphology. Several reasons may underlie the greater sensitivity of primary midbrain neurons, as opposed to astrocytes, to death via oxidative inactivation of m-aconitase. The substantia nigra has been demonstrated to have low glutathione levels relative to other brain regions. In PD, levels are still further decreased leading to inefficient removal of H_2_O_2_ from this region of the brain [40], [41]. In addition to low glutathione levels, additional evidence for oxidative stress in PD comes from studies showing increased iron levels and antioxidant imbalance [42], [43]. However, because astrocytes maintain a high glutathione-glutathione peroxidase content, they are generally protected [25]. Collectively, these reasons may render midbrain neurons more sensitive to neuronal death via oxidative inactivation of m-aconitase.

“Cross-talk” between astrocyte-derived H_2_O_2_ and neurons may be an important mechanism for neuronal injury, especially in diseases like PD where neurotoxicity is concomitant with astrogliosis and inflammation [44], [45]. Astrogliosis may increase the amount of m-aconitase available for oxidative inactivation. This event could potentially increase astrocyte-derived H_2_O_2_ production and lead to injury of neighboring neurons. This may hold especially true in neurological disorders where an environment of oxidative stress persists, as is observed with many neurodegenerative disorders.

In summary, this study implicates m-aconitase as a source of H_2_O_2_ and Fe^2+^ and as a key contributor to cell death in neuronal cells. The findings also confirm the notion that therapeutic intervention with iron chelation and/or mitochondrial antioxidants may be potentially useful in neuronal diseases where mitochondrial oxidative stress predominates.
